# Granulocyte colony-stimulating factors for febrile neutropenia prophylaxis following chemotherapy: systematic review and meta-analysis

**DOI:** 10.1186/1471-2407-11-404

**Published:** 2011-09-23

**Authors:** Katy L Cooper, Jason Madan, Sophie Whyte, Matt D Stevenson, Ron L Akehurst

**Affiliations:** 1 School of Health and Related Research (ScHARR), University of Sheffield, Sheffield, UK; 2 Academic Unit of Primary Health Care, University of Bristol, Bristol, UK

## Abstract

**Background:**

Febrile neutropenia (FN) occurs following myelosuppressive chemotherapy and is associated with morbidity, mortality, costs, and chemotherapy reductions and delays. Granulocyte colony-stimulating factors (G-CSFs) stimulate neutrophil production and may reduce FN incidence when given prophylactically following chemotherapy.

**Methods:**

A systematic review and meta-analysis assessed the effectiveness of G-CSFs (pegfilgrastim, filgrastim or lenograstim) in reducing FN incidence in adults undergoing chemotherapy for solid tumours or lymphoma. G-CSFs were compared with no primary G-CSF prophylaxis and with one another. Nine databases were searched in December 2009. Meta-analysis used a random effects model due to heterogeneity.

**Results:**

Twenty studies compared primary G-CSF prophylaxis with no primary G-CSF prophylaxis: five studies of pegfilgrastim; ten of filgrastim; and five of lenograstim. All three G-CSFs significantly reduced FN incidence, with relative risks of 0.30 (95% CI: 0.14 to 0.65) for pegfilgrastim, 0.57 (95% CI: 0.48 to 0.69) for filgrastim, and 0.62 (95% CI: 0.44 to 0.88) for lenograstim. Overall, the relative risk of FN for any primary G-CSF prophylaxis versus no primary G-CSF prophylaxis was 0.51 (95% CI: 0.41 to 0.62). In terms of comparisons between different G-CSFs, five studies compared pegfilgrastim with filgrastim. FN incidence was significantly lower for pegfilgrastim than filgrastim, with a relative risk of 0.66 (95% CI: 0.44 to 0.98).

**Conclusions:**

Primary prophylaxis with G-CSFs significantly reduces FN incidence in adults undergoing chemotherapy for solid tumours or lymphoma. Pegfilgrastim reduces FN incidence to a significantly greater extent than filgrastim.

## Background

Neutropenia is the major dose-limiting toxicity of many chemotherapy regimens. Grade 3 and grade 4 neutropenia are defined as a neutrophil count < 1.0 × 10^9^/L and < 0.5 × 10^9^/L respectively. Febrile neutropenia (FN) is defined as neutropenia with fever, usually indicating infection, and is associated with substantial morbidity, mortality, and costs [1]. The direct risk of mortality associated with FN has been estimated as 9.5% (95% confidence interval [CI]: 9.2%, 9.8%) in a study of 41,779 cancer patients hospitalised with FN [1]. Management of FN often requires lengthy hospitalisation, [1] with associated costs and detrimental effects on quality of life [2,3]. In addition, an FN episode has been shown to increase the risk of chemotherapy dose reductions and delays [4]. Unplanned reductions in chemotherapy dose may cause further deaths from cancer in the long-term; in a retrospective analysis of breast cancer patients with a 30-year follow-up, the survival rate was 40% (95% CI: 26%, 55%) among patients receiving at least 85% of their planned dose, but only 21% (95% CI: 14%, 26%) among patients who received less than 85% [5].

Recombinant human granulocyte colony-stimulating factors (G-CSFs) stimulate production of mature, functional neutrophils [6]. G-CSFs have been shown to reduce the incidence of FN when used as prophylaxis following chemotherapy. Three G-CSFs are currently in common usage: filgrastim, pegfilgrastim, and lenograstim. Filgrastim and lenograstim are administered as a series of daily injections; clinical studies suggest an average of 11 injections per chemotherapy cycle are required to achieve recovery of the absolute neutrophil count (ANC) to within the normal range [7-10]. Pegfilgrastim is administered as a single injection per chemotherapy cycle [11,12]. G-CSFs may be administered as primary prophylaxis (in every chemotherapy cycle from cycle 1) or as secondary prophylaxis (in all remaining cycles following a neutropenic event such as FN or prolonged severe neutropenia). The overall FN risk is dependent on chemotherapy regimen as well as individual patient risk factors such as age, performance status and disease stage [13]. Guidelines from the European Organisation for Research and Treatment of Cancer (EORTC), [13] the American Society of Clinical Oncology (ASCO) [14] and the National Comprehensive Cancer Network (NCCN) [15] recommend that prophylactic G-CSFs should be used where the risk of FN associated with the chemotherapy regimen is greater than or equal to 20%, and may be considered where the risk is 10-20%, particularly where additional patient risk factors are present.

This paper reports a systematic review and meta-analysis of the effect of primary G-CSF prophylaxis (with pegfilgrastim, filgrastim or lenograstim) on incidence of FN. The effect of each G-CSF is assessed in comparison with no primary G-CSF prophylaxis and in comparison with other G-CSFs.

## Methods

### Search strategy

The systematic review followed the recommendations in the PRISMA statement [16,17]. A systematic search was undertaken to identify randomised controlled trials (RCTs) of pegfilgrastim, filgrastim or lenograstim, compared with no primary G-CSF or with one another, for the reduction of FN following chemotherapy. A previous systematic review by Kuderer et al. [18] presented a meta-analysis of FN incidence within RCTs of primary G-CSF prophylaxis versus no primary G-CSF prophylaxis, while a systematic review by Pinto et al. [19] meta-analysed RCTs of primary prophylaxis using pegfilgrastim versus filgrastim. The literature searches within these previous reviews were conducted during 2006. Therefore, databases were searched from 2006 onwards, whereas studies published prior to 2006 were identified from the two existing reviews. Searches were undertaken in December 2009. The following databases were searched: Medline, Medline in Process, EMBASE, Science Citation Index, Cochrane Database of Systematic Reviews, Cochrane CENTRAL Register of Controlled Trials, Database of Abstracts of Reviews of Effects (DARE), Health Technology Assessment Database and NHS Economic Evaluation Database (NHS-EED). The Medline search strategy was designed with reference to the previous two reviews, and comprised subject headings and text words for G-CSFs combined with a search filter to identify RCTs (Appendix 1). Searches were not restricted by language. Bibliographies of retrieved papers were searched for any additional relevant studies.

### Inclusion and exclusion criteria

Studies were considered suitable for inclusion if they assessed primary G-CSF prophylaxis (pegfilgrastim, filgrastim or lenograstim) administered 1-3 days after the completion of chemotherapy, versus a different G-CSF or versus no primary G-CSF prophylaxis. Studies were only included if they reported incidence of FN. For consistency with the two existing systematic reviews, [18,19] only studies of adult cancer patients with solid tumours or lymphoma were included. Studies allowing concomitant antibiotic prophylaxis were included if identical prophylaxis was administered in both study arms. The following study types were excluded: studies of G-CSFs for treatment of FN; studies in children; studies in patients with leukaemia, myeloid malignancies or myelodysplastic syndromes; studies of G-CSFs for stem cell mobilisation in bone marrow or peripheral blood stem cell transplantation; economic analyses; studies with differing drugs, doses or schedules of chemotherapy in each arm; studies with differing doses of the same G-CSF in each arm; and studies not published in English.

### Outcome measures

The outcome measure assessed in this review was the incidence of FN over all cycles of chemotherapy within each study. FN was chosen as a key clinical outcome due to its direct bearing on morbidity, mortality and hospitalisation rates, and also because this review was undertaken alongside the development of an economic model which utilised FN rate as a key parameter.

### Data extraction

Data was extracted by two reviewers using a form developed for this review and any discrepancies were resolved through discussion.

### Data synthesis

Meta-analyses were undertaken to compare the effectiveness of G-CSFs versus no prophylaxis and versus each other for the reduction of FN. Analyses were undertaken using RevMan software (version 5, Cochrane Collaboration). Results for each comparison were presented as a pooled relative risk and 95% CIs. Although clinical and statistical heterogeneity existed between studies, there was insufficient data on individual populations to facilitate separate analyses. Therefore, for consistency with existing reviews, all studies were included in the analysis, and a random effects model was used. Heterogeneity was presented using the I^2 ^statistic, which describes the percentage of the variability in effect estimates that is due to heterogeneity rather than sampling error (chance) [20].

## Results

### Number and characteristics of included studies

**Figure 1:**
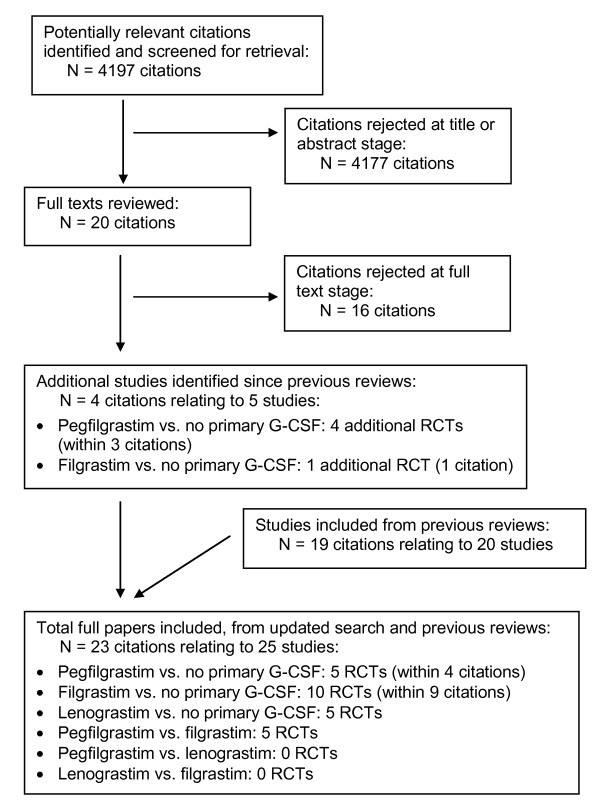
**Flow chart for identification of relevant studies**.

The flow chart for study inclusion is shown in Figure 1 and the included studies are described in Table 1. Studies published from 2006 onwards were identified from the literature search, and studies published prior to 2006 were identified from two previous reviews [18,19]. In total, 23 citations relating to 25 studies satisfied the inclusion criteria: 5 studies of primary pegfilgrastim vs. no primary G-CSF (within 4 citations); [21-24] 10 studies of primary filgrastim vs. no primary G-CSF (within 9 citations); [25-33] 5 studies of primary lenograstim vs. no primary G-CSF; [9,10,34-36] and 5 studies of primary pegfilgrastim vs. primary filgrastim [7,8,37-39]. No studies were identified comparing lenograstim with either pegfilgrastim alone or filgrastim alone.

**Table 1:** Description of trials of primary G-CSFs (vs. no primary G-CSF, or vs. each other)

Trial	Study design	Cancer type	Cancer stage	Patient age	Chemotherapy regimen	N cycles (max)	Cycle length	Arm 1 G-CSF strategy ^b^	Arm 1: N analysed	Arm 1: days primary G-CSF	Arm 2 G-CSF strategy ^b^	Arm 2: N analysed	Arm 2: days primary G-CSF	FN definition
**Pegfilgrastim** **vs. no primary** **G-CSF**														

^a ^Romieu 2007[23]	RCT, phase II, OL	Breast cancer	Stage II-III, node-positive	Age ≥ 65. Median 68, range 65-77	FEC-100	6 (FN reported cycle 1 only)	3 weeks	Pegfilgrastim primary: 6 mg day 2	30	1	No primary G-CSF, pegfilgrastim secondary (following FN or neutropenia)	29	0	Fever + ANC < 1 × 10^9^/l

Vogel 2005[21]	RCT, phase III, DB	Breast cancer	62% stage IV, 38% other stages	Mean age 52, range 21-88	Docetaxel 100 mg/m^2^	4	3 weeks	Pegfilgrastim primary: 6 mg day 2	463	1	Placebo primary, pegfilgrastim secondary (following FN)	465	0	Fever + ANC < 0.5 × 10^9^/l

^a ^Hecht 2009[24]	RCT, phase II	Colorectal cancer	NR	NR	FOLFOX (49%), FOLFIRI (26%) or FOIL (25%)	4	2 weeks	Pegfilgrastim primary: 6 mg day 4	123	1	Placebo primary	118	0	Grade 3-4 FN (assumed fever + ANC < 1 × 10^9^/l)

^a ^Balducci 2007: NHL[22]	RCT, OL	NHL	38% stage I-II, 62% stage III-IV	Age ≥ 65. Median 72, range 65-88	CHOP or R-CHOP	6	3 weeks	Pegfilgrastim primary: 6 mg day 2	73	1	No primary G-CSF, pegfilgrastim secondary (at physician's discretion)	73	0	Fever + ANC < 1 × 10^9^/l

^a ^Balducci 2007: solid tumour[22]	RCT, OL	Solid tumour (lung, ovarian, breast)	31% stage I-II, 69% stage III-IV	Age ≥ 65. Median 72, range 65-88	One of 15 regimens with mild-to-moderate risk of neutropenia	6	3 weeks	Pegfilgrastim primary: 6 mg day 2	343	1	No primary G-CSF, pegfilgrastim secondary (at physician's discretion)	343	0	Fever + ANC < 1 × 10^9^/l

**Filgrastim vs. no primary** **G-CSF**														

^a ^del Giglio 2008[33]	RCT, DB	Breast cancer	21% high-risk stage II, 53% stage III, 25% stage IV	Mean age 51, range 25-75	Doxorubicin 60 mg/m^2^/docetaxel 75 mg/m^2^	4 (FN reported cycle 1 only)	3 weeks	Filgrastim primary (Neupogen or XM02): 5 ug/kg/d from day 2 up to 14d or to ANC = 10 × 10^9^/l	276	5 to 14 (median 9-10)	Placebo in cycle 1; filgrastim (XM02) in subsequent cycles	72	0 (cycle 1)	Fever + ANC < 0.5 × 10^9^/l

Timmer-Bonte 2005[29]	RCT, phase III, OL	SCLC	69% extensive, 31% limited	Age range 36-81	CDE	5	3 weeks	Filgrastim primary: 300/450 ug/d from day 4; prophylactic antibiotics	90	10	No primary G-CSF; prophylactic antibiotics	85	0	Fever + ANC < 0.5 × 10^9^/l

Trillet-Lenoir 1993[30]	RCT, DB	SCLC	64% extensive, 36% limited	Median 59	CDE	6	3 weeks	Filgrastim primary: 230 ug/m^2^/d from day 4 up to 14d or until ANC = 10 × 10^9^/l	65	9 to 14	Placebo primary	64	0	Fever + ANC < 1 × 10^9^/l

Crawford 1991[31]	RCT, DB	SCLC	72% extensive, 28% limited	Age range 31-80	CDE	6	3 weeks	Filgrastim primary: 230 ug/m^2^/d from day 4 up to 14d or until ANC = 10 × 10^9^/l	95	9 to 14	Placebo primary; secondary G-CSF	104	0	Fever + ANC < 1 × 10^9^/l

Doorduijn 2003[25]	RCT, OL	Aggressive NHL	Stage II-IV	Age ≥ 65. Median 72, range 65-90	CHOP	6 to 8	3 weeks	Filgrastim primary: 300 ug/d from day 2 for 10d	197	10	No primary G-CSF	192	0	FN not defined in terms of ANC

Osby 2003 (C**H**OP)[26]	RCT, OL	Aggressive NHL	Stage II-IV	Age ≥ 60. Median 71, range 60-86	CHOP	4 to 8 (most 8)	3 weeks	Filgrastim primary: 5 ug/kg/d from day 2 up to 14d or until ANC = 10 × 10^9^/l	101	10 to 14	No primary G-CSF	104	0	Fever + ANC < 0.5 × 10^9^/l

Osby 2003 (C**N**OP)[26]	RCT, OL	Aggressive NHL	Stage II-IV	Age ≥ 60. Median 71, range 60-86	CNOP	4 to 8 (most 8)	3 weeks	Filgrastim primary: 5 ug/kg/d from day 2 up to 14d or until ANC = 10 × 10^9^/l	125	10 to 14	No primary G-CSF	125	0	Fever + ANC < 0.5 × 10^9^/l

Zinzani 1997[27]	RCT, OL	Aggressive NHL	Stage II-IV	Age ≥ 60. Age range 60-82	VNCOP-B	8	1 week (differs alternate weeks)	Filgrastim primary: 5 ug/kg/d from day 3; prophylactic antibiotics	77	5	No primary G-CSF; prophylactic antibiotics	72	0	FN not defined in terms of ANC

Pettengell 1992[28]	RCT, OL	Aggressive NHL	Any stage	Age range 16-71	VAPEC-B	11	1 week (differs alternate weeks)	Filgrastim primary: 230 ug/m^2^/d from day 2 up to 14d or until ANC = 10 × 10^9^/l; prophylactic antibiotics	41	12	No primary G-CSF; prophylactic antibiotics	39	0	Fever + ANC < 1 × 10^9^/l

Fossa 1998[32]	RCT, phase III, OL	Germ cell cancer	Metastatic, poor-prognosis	Age range 15-65	BEP/EP or BOP/VIP-B	6	3 weeks or 10 d	Filgrastim primary: 5 ug/kg/d from day 3 or 6	129	7 or 14	No primary G-CSF	130	0	FN not defined in terms of ANC

**Lenograstim vs. no primary G-CSF**														

Chevallier 1995[9]	RCT, DB	Breast cancer, inflammatory	Non-metastatic	Age range 23-65	FEC-high-dose	4	3 weeks	Lenograstim primary: 5 ug/kg/d from day 6	61	10	Placebo primary	59	0	Fever + ANC < 1 × 10^9^/l

Gisselbrecht 1997[34]	RCT, DB	Aggressive NHL	Any stage	Age range 15-55	LNH-87 (LNH-84 + randomization to anthracyclines)	4	2 weeks	Lenograstim primary: 5 ug/kg/d from day 6	82	8	Placebo primary	80	0	Fever + ANC < 1 × 10^9^/l

Bui 1995[10]	RCT, DB	Soft tissue sarcoma	Metastatic or locally advanced	Age range 21-69	MAID	6 (FN reported cycle 1 only)	3 weeks	Lenograstim primary: 5 ug/kg/d from day 4 up to 14d or until ANC = 30 × 10^9^/l	22	10 to 14	Placebo primary; secondary G-CSF	26	0	Fever + ANC < 1 × 10^9^/l

Gebbia 1994[35]	RCT, DB	Various	Advanced	Age range 40-75	Various	Various	Various	Lenograstim primary: 5 ug/kg/d	23	≥ 7d	Placebo primary	28	0	Fever + ANC < 1 × 10^9^/l

Gebbia 1993[36]	RCT, DB	Various	Advanced	Age range 38-66	Various	Various	Various	Lenograstim primary: 5 ug/kg/d	43	7 to 10	Placebo primary	43	0	Fever + ANC < 1 × 10^9^/l

**Pegfilgrastim vs. 10- or 11-day filgrastim**														

Green 2003[7]	RCT, phase III, DB	Breast cancer	28% stage II, 27% stage III, 45% stage IV	Mean age 52, range 30-75	Doxorubicin 60 mg/m^2^/docetaxel 75 mg/m^2^	4	3 weeks	Pegfilgrastim primary: 6 mg day 2; then placebo up to 14d	77	1	Filgrastim primary: 5 ug/kg, from day 2 up to 14d or until ANC = 10 × 10^9^/l	75	11 (median)	Fever + ANC < 0.5 × 10^9^/l

Holmes 2002: phase III[8]	RCT, phase III, DB	Breast cancer	High-risk stage II, III or IV. 37% stage IV	Mean age 51	Doxorubicin 60 mg/m^2^/docetaxel 75 mg/m^2^	4	3 weeks	Pegfilgrastim primary: 100 ug/kg day 2; then placebo up to 14d	149	1	Filgrastim primary: 5 ug/kg, from day 2 up to 14d or until ANC = 10 × 10^9^/l	147	11 (mean)	Fever + ANC < 0.5 × 10^9^/l

Holmes 2002: phase II[37]	RCT, phase II, DF	Breast cancer	High-risk stage II, III or IV. 30% stage IV	Mean age 49	Doxorubicin 60 mg/m^2^/docetaxel 75 mg/m^2^	4	3 weeks	Pegfilgrastim primary: 100 ug/kg day 2 (other dose groups not included here)	46	1	Filgrastim primary: 5 ug/kg, from day 2 up to 14d or until ANC = 10 × 10^9^/l	25	10.6; 10.2; 10.4; 11.0 (mean in cycles 1-4)	Fever + ANC < 0.5 × 10^9^/l

Grigg 2003[38]	RCT, phase II, OL, DF	NHL	Any stage	Age ≥ 60. Mean 68, range 60-82	CHOP	6	3 weeks	Pegfilgrastim primary: 100 ug/kg day 2 (other dose groups not included here)	14	1	Filgrastim primary: 5 ug/kg, from day 2 up to 14d or until ANC = 10 × 10^9^/l	13	10 (mean)	Fever + ANC < 0.5 × 10^9^/l

Vose 2003[39]	RCT, phase II, OL	NHL (n = 56) or HL (n = 4)	Relapsed or refractory	Mean age 49. 85% < 65	ESHAP	4 (FN reported cycles 1 & 2 only)	3 weeks	Pegfilgrastim primary: 100 ug/kg day 2	29	1	Filgrastim primary: 5 ug/kg, from day 2 up to 12d or until ANC = 10 × 10^9^/l	31	11 (median)	Fever + ANC < 0.5 × 10^9^/l

^a ^Studies added as a result of updated search. ^b ^G-CSF strategy: Primary prophylaxis is in all cycles. Secondary prophylaxis is in all cycles following FN, or following FN or neutropenia, or at physician's discretion (as noted for individual studies). ANC = absolute neutrophil count; DB = double-blind; DF = dose-finding; HL = Hodgkin's lymphoma; NHL = non-Hodgkin's lymphoma; OL = open-label; SCLC = small-cell lung cancer. Chemotherapy regimens used: BEP/EP = etoposide 100 mg/m^2^, cisplatin 20 mg/m^2^, plus or minus bleomycin 30 U. BOP/VIP-B = bleomycin 30 U, vincristine 2 mg, cisplatin 20-50 mg/m^2^/etoposide 100 mg/m^2^, ifosfamide 1000 mg/m^2^. CDE = cyclophosphamide 1 g/m^2^, doxorubicin 45-50 mg/m^2^, etoposide 100-120 mg/m^2^. CHOP = cyclophosphamide 750 mg/m^2^, doxorubicin 50 mg/m^2^, vincristine 1.4 mg/m^2^, prednisolone 100 mg days 1-5. CNOP = cyclophosphamide 750 mg/m^2^, mitoxantrone 10 mg/m^2^, vincristine 1.4 mg/m^2^, prednisolone 50 mg/m^2 ^days 1-5. ESHAP = etoposide 40 mg/m^2^, methylprednisolone 500 mg, cisplatin 25 mg/m^2^/d, cytarabine 2000 mg/m^2^. FEC-100 = 5-fluorouracil 500 mg/m^2^, epirubicin 100 mg/m^2^, cyclophosphamide 500 mg/m^2^. FEC-high-dose = 5-fluorouracil 750 mg/m^2^, epirubicin 35 mg/m^2^, cyclophosphamide 400 mg/m^2^. FOIL = 5-FU, oxaliplatin, irinotecan, leucovorin. FOLFIRI = 5-FU, irinotecan, leucovorin. FOLFOX = 5-FU, oxaliplatin, leucovorin. LNH-87 = cyclophosphamide 1200 mg/m^2 ^day 1, vindesine 2 mg/m^2 ^days 1 & 5, bleomycin 10 mg days 1 & 5, prednisolone 60 mg/m^2 ^days 1-5, methotrexate 15 mg, with either doxorubicin 75 mg/m^2 ^or mitoxantrone 12 mg/m^2 ^day 1. MAID = mesna, doxorubicin, ifosfamide, dacarbazine. R-CHOP = CHOP plus rituximab. TAC = doxorubicin 50 mg/m^2^, cyclophosphamide 500 mg/m^2^, docetaxel 75 mg/m^2^. VAPEC-B = vincristine 1.4 mg/m^2^, doxorubicin 35 mg/m^2 ^prednisolone 50 mg/d (then tapered), etoposide 100 mg/m^2^, cyclophosphamide 350 mg/m^2^, bleomycin 10 mg/m^2^. VNCOP-B = vincristine 2 mg, mitoxantrone 10 mg/m^2^, cyclophosphamide 300 mg/m^2^, etoposide 150 mg/m^2^, prednisone 40 mg, bleomycin 10 mg/m^2^.

A previous systematic review of prophylactic G-CSF use [18] included only a single study of pegfilgrastim versus no primary G-CSF [21]. Our literature search identified 4 additional RCTs of pegfilgrastim vs. no primary G-CSF, which were conducted in populations with colorectal cancer, [24] breast cancer, [23] non-Hodgkin's lymphoma, [22] and various solid tumours; [22] the latter three studies were restricted to elderly patients. Our review also identified an additional large RCT of filgrastim vs. no primary G-CSF in breast cancer [33].

There was heterogeneity among trials of all three G-CSFs in terms of cancer type, patient age, chemotherapy regimen, number of chemotherapy cycles and cycle length (Table 1). Filgrastim and lenograstim were generally given for 10-14 days where the chemotherapy cycle length was 3 weeks (and for fewer days in a small number of trials with shorter cycle lengths). The comparator arm in some of the studies included secondary G-CSFs for those patients having an FN event, and some trials allowed prophylactic antibiotics in both arms. Some studies were open-label rather than double-blind.

### Effectiveness of G-CSFs in reducing febrile neutropenia incidence

**Figure 2:**
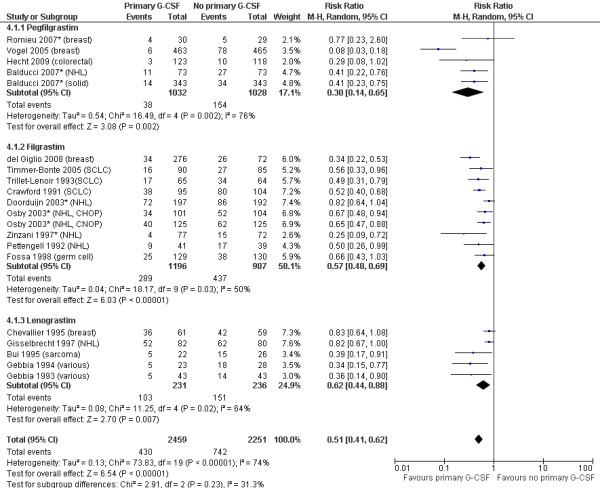
**Primary G-CSFs versus no primary G-CSF: FN incidence**. Cancer types for each study are shown after the author and date. CHOP and CNOP = chemotherapy regimens for NHL (see Table 1 footnote); NHL = non-Hodgkin's lymphoma; SCLC = small-cell lung cancer; solid = solid tumours. *Indicates studies in patients aged ≥ 60 or ≥ 65 years.

**Figure 3:**
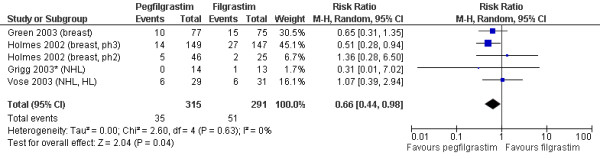
**Pegfilgrastim versus filgrastim: FN incidence**. Cancer types for each study are shown after the author and date. HL = Hodgkin's lymphoma; NHL = non-Hodgkin's lymphoma. *Indicates studies in patients aged ≥ 60 or ≥ 65 years. In the Holmes 2002 (phase II) study,[37] FN incidence in the filgrastim arm was reported as 2/25, which was incorrectly converted to 12%. The absolute numbers (2/25) have been used in this analysis. Therefore the resulting relative risk differs slightly from that reported in the previous systematic review by Pinto (2007),[19] which used the 12% figure.

The relative risks of FN incidence are shown in Figure 2 for trials of G-CSF versus no primary G-CSF, and in Figure 3 for trials of pegfilgrastim versus filgrastim. The pooled relative risks for each G-CSF comparison are summarised in Table 2. Primary prophylaxis with each of the G-CSFs significantly decreased the risk of FN compared with no primary G-CSF, with relative risks of 0.30 (95% CI: 0.14 to 0.65) for pegfilgrastim, 0.57 (95% CI: 0.48 to 0.69) for filgrastim, and 0.62 (95% CI: 0.44 to 0.88) for lenograstim. Overall, the relative risk of FN when using any primary G-CSF prophylaxis versus no primary G-CSF prophylaxis was 0.51 (95% CI: 0.41 to 0.62).

**Table 2:** Summary of febrile neutropenia incidence based on meta-analyses of trials of G-CSFs

Treatment 1	Treatment 2	No of studies	No of patients	Relative risk of FN (95% CI)	p-value	I^2 ^(heterogeneity)
Pegfilgrastim	No primary G-CSF	5	2060	0.30 (0.14 to 0.65)	p = 0.002	76%

Filgrastim	No primary G-CSF	10	2183	0.57 (0.48 to 0.69)	p < 0.00001	50%

Lenograstim	No primary G-CSF	5	467	0.62 (0.44 to 0.88)	p = 0.007	64%

Any G-CSF	No primary G-CSF	20	4710	0.51 (0.41 to 0.62)	p < 0.00001	74%

Pegfilgrastim	Filgrastim	5	606	0.66 (0.44 to 0.98)	p = 0.04	0%

There was a relatively high level of statistical heterogeneity in the analyses as shown by the I^2 ^statistic, which ranged from 50-76%; this is likely to reflect the variations between studies in factors such as cancer type, patient age, chemotherapy regimen, number of chemotherapy cycles and cycle length. Individual studies differed on too many variables for formal sub-analyses to be meaningful. However, Figures 2 and 3 illustrate the cancer type for each study (shown after the author and date) and highlight studies in populations aged ≥ 60 or ≥ 65 years (shown by asterisks). There was no clear difference in G-CSF effectiveness between cancer types, nor in studies restricting to an elderly population. As the majority of studies administered filgrastim and lenograstim for 10-14 days (for 3-week chemotherapy cycles), there was insufficient data to assess the effects of shorter durations of G-CSF treatment.

In terms of comparisons between different G-CSFs, the relative risk of FN for pegfilgrastim versus filgrastim was 0.66 (95% CI: 0.44 to 0.98). There were no head-to-head trials comparing lenograstim to either of the other two G-CSFs.

## Discussion

Our systematic review and meta-analyses confirm and strengthen previous evidence that primary prophylaxis with each of the three G-CSFs is effective in reducing the risk of FN following chemotherapy. In particular, our systematic review identified 4 further RCTs of pegfilgrastim vs. no primary G-CSF, [22-24] whereas at the time of a previous systematic review [18] only a single RCT [21] making this comparison was available. Although these 5 RCTs comparing pegfilgrastim with no primary G-CSF were heterogeneous in terms of clinical population and chemotherapy regimen, the pooled relative risk indicated a significant effect of pegfilgrastim in reducing FN incidence. Filgrastim and lenograstim also significantly reduced FN incidence.

This review also strengthens the evidence base regarding the comparative effectiveness of the three G-CSFs; in particular, comparison of the "once-per-cycle" G-CSF pegfilgrastim versus the "once-daily" G-CSF filgrastim. Meta-analysis of five RCTs indicated that FN incidence was significantly lower following primary prophylaxis with pegfilgrastim than with filgrastim. This is consistent with the fact that the reduction in FN risk for pegfilgrastim versus no primary G-CSF was greater than the reduction observed for filgrastim versus no primary G-CSF.

As discussed in previous reviews, [18,19] there was heterogeneity among the studies in terms of the clinical population (age, cancer type), chemotherapy regimen, and cycle length and number. Correspondingly, heterogeneity was observed among the study results. Since individual studies differed on too many variables for formal sub-analyses to be meaningful, all studies were included in the analysis. There was no clear difference in G-CSF effectiveness between cancer types, nor in studies restricting to elderly populations. However, the variation in clinical population, and the corresponding high levels of heterogeneity, indicate that caution should be used when applying the results to individual clinical settings. Conversely, the range of populations and treatment regimens covered by the included studies is likely to reflect the variations which would be observed in clinical practice.

## Conclusions

This systematic review and meta-analysis demonstrate that primary G-CSF prophylaxis with pegfilgrastim, filgrastim and lenograstim is effective in reducing the risk of FN in adults undergoing chemotherapy for solid tumours or lymphoma. In addition, although heterogeneity existed between studies, a meta-analysis suggests that pegfilgrastim reduces the risk of FN to a greater extent than filgrastim.

## List of abbreviations

ANC: Absolute neutrophil count; ASCO: American Society of Clinical Oncology; DARE: Database of Abstracts of Reviews of Effects; EORTC: European Organisation for Research and Treatment of Cancer; FN: Febrile neutropenia; G-CSF: Granulocyte colony-stimulating factor; NCCN: National Comprehensive Cancer Network; NHS-EED: NHS Economic Evaluation Database; RCT: Randomised controlled trial.

## Competing interests

The research underlying this paper was funded by Amgen Ltd, and a research grant from Amgen (EUROPE) GmbH was provided to support the production of the manuscript. Amgen staff reviewed and made suggested edits to the manuscript, but final content, authorship and right to publication remained with the research team.

## Authors' contributions

KC undertook the systematic review and drafted the manuscript. JM undertook the statistical analyses. SW contributed to study selection and interpretation. MS contributed to study selection and interpretation and to the statistical analyses. RA participated in the design and coordination of the study and contributed to the statistical analyses. All authors read and approved the final manuscript.

## Appendix 1: Search strategy (Medline)

1 Granulocyte colony-stimulating factor/

2 Granulocyte colony-stimulating factor, recombinant/

3 Colony-stimulating factors, recombinant/

4 Filgrastim/

5 G-CSF$

6 granulocyte colony-stimulating factor$

7 filgrastim

8 Neupogen

9 pegfilgrastim

10 Neulasta

11 lenograstim

12 Granocyte

13 Euprotin

14 r-metHuG-CSF

15 SD-01

16 PEG-rmetHuG-CSF

17 XM02

18 Ratiograstim

19 or/1-18

20 randomized controlled trial.pt.

21 controlled clinical trial.pt.

22 randomized controlled trial/

23 random allocation/

24 double blind method/

25 single blind method/

26 clinical trial.pt.

27 exp clinical trial/

28 (clin$ adj25 trial$).ti,ab.

29 ((singl$ or doubl$ or trebl$ or tripl$) adj25 (blind$ or mask$)).ti,ab.

30 placebos/

31 placebos.ti,ab.

32 random.ti,ab.

33 research design/

34 randomised.ti,ab

35 randomized.ti,ab

36 or/20-35

37 19 and 36

("$" indicates truncations; "/" indicates medical subject headings)

## Pre-publication history

The pre-publication history for this paper can be accessed here:

http://www.biomedcentral.com/1471-2407/11/404/prepub
